# Between automation and alienation: rethinking AI’s role in radiologist well-being

**DOI:** 10.1007/s00330-026-12473-z

**Published:** 2026-03-20

**Authors:** Elif Can, Jawed Nawabi

**Affiliations:** 1 Department of Diagnostic and Interventional Radiology, Medical Center – University of Freiburg, Faculty of Medicine, University of Freiburg, Hugstetter Str. 55, 79106, Freiburg im Breisgau, Germany; 2 Department of Neuroradiology, Charité–Universitätsmedizin Berlin, Corporate Member of Freie Universität Berlin and Humboldt Universität zu Berlin, Berlin, Germany

Radiology has the highest degree of AI exposure across clinical specialties. As of late 2025, approximately 80% of FDA-cleared AI-enabled medical devices target radiological applications [1]. Despite this rapid adoption, evidence on how AI affects radiologist well-being remains fragmented. While AI promises efficiency gains, few studies systematically assess its net impact on workload, autonomy/control, professional meaning, or moral injury factors that shape both well-being and burnout. A recent cross-sectional study among radiologists in China identified a positive correlation between frequent AI use and burnout, especially in contexts of high workload and low acceptance of AI systems [2]. Burnout is a key adverse outcome within professional well-being [3].

This concept advances the core thesis that AI is neither inherently protective nor harmful regarding burnout. Rather, its effects are shaped by implementation design, governance, and the sociotechnical environment. This view aligns with burnout literature, which conceptualizes physician burnout primarily as a systemic problem, not a failure of individual resilience. Accordingly, burnout mitigation strategies must operate on multiple levels, from organizational culture (e.g., reducing unnecessary documentation) to structural reform [3, 4].

Therefore, AI’s effects on radiologist well-being should be appraised through established system-level determinants of professional well-being, commonly operationalized in the burnout literature (workload, autonomy/control, work-life integration, social support, organizational culture, and efficiency/resources), rather than assuming that automation alone is protective, thereby framing AI as either a buffer or an amplifier of existing stressors [5].

## AI as a potential buffer

When properly implemented, AI can reduce burnout, but only if it lowers net workload and net cognitive burden, rather than shifting work into ‘supervision mode’ [5]. If radiologists must continuously verify AI-generated documentation or monitor algorithmic outputs for errors, the promised time savings can translate into added vigilance, interruption costs, and mental load. Burnout mitigation depends on reducing true burden without converting work into continuous verification or eliminating restorative low-load intervals [3].

If AI compresses work into uninterrupted high-load interpretation plus constant oversight, departments should compensate through deliberate pacing measures (e.g., protected breaks, workload caps, or task redistribution). In mammography, AI-assisted workflows have been reported to reduce workload without compromising diagnostic performance, but gains depend on workflow placement, adjudication burden, and whether time savings translate into real relief rather than higher throughput [6].

## AI as a burnout amplifier

However, AI may intensify burnout if it exacerbates systemic stressors. Efficiency gains can be reappropriated as productivity targets, leading to increased throughput and time pressure without offsetting workload reductions. Additional risks include cognitive overload (e.g., excessive alerts, false positives), documentation redundancies, and role ambiguity (e.g., unclear accountability for AI-generated errors). A growing body of evidence suggests that under-resourced institutions may deploy AI in ways that inadvertently increase rather than decrease workload [5, 7]. Beyond workload escalation, AI may also subtly reshape professional autonomy. When clinical performance becomes increasingly defined by measurable outputs, such as throughput, error rates, or concordance with algorithmic predictions, radiological expertise risks being reframed as metric compliance rather than contextual clinical judgment [8]. Radiologists may remain accountable for outcomes while having limited influence over workflow tempo, evaluative standards, or implementation design.

Legal and accountability ambiguities may further intensify stress. When AI-generated outputs inform clinical decisions, unclear responsibility for diagnostic errors can create defensive practice patterns and moral distress. Radiologists may absorb the cognitive and legal remainder when systems fail, despite limited control over algorithm design or validation processes.

## Ideological overload and “AI-washing”

AI is frequently marketed as a neutral and inevitable solution to workforce challenges. However, unsubstantiated promises of relief can backfire when they fail to materialize. Discrepancies between techno-optimistic narratives and clinical realities can contribute to moral injury, i.e., the distress caused by conflict between institutional constraints and professional values. Critical voices have thus called for tempered expectations and evidence-based implementation pathways [7]. In competitive healthcare environments, AI adoption may also be driven by reputational or strategic incentives rather than demonstrated clinical benefit. Institutions may feel compelled to signal innovation to remain attractive to patients, investors, or policymakers. When symbolic adoption precedes evidence-based integration, implementation fatigue and erosion of trust can follow. Overpromising and underdelivering may foster cynicism and contribute to moral injury when expectations of relief conflict with everyday clinical realities [9].

## Systemic pressures and governance

Economic incentives, workforce shortages, and output-driven management structures strongly influence how AI is deployed in radiology. Across Europe, demand for imaging services continues to grow, while many countries face chronic shortages of trained radiologists [4]. In such a strained environment, the introduction of complex, often insufficiently regulated AI tools may further exacerbate operational and cognitive burdens rather than alleviate them. From a broader sociotechnical perspective, AI implementation may reflect a shift toward quantification and optimization as increasingly dominant organizing principles in healthcare. If left unexamined, such logics may unintentionally narrow professional discretion and redefine clinical value primarily in terms of measurable performance.

Without protective governance frameworks, automation risks becoming a mechanism for labor intensification and value extraction.

If AI-derived metrics are incorporated into performance evaluation or productivity benchmarking without clinician involvement, this may further narrow professional discretion and reinforce throughput-driven management logics [9].

Studies suggest that efficiency gains from AI are more likely to accrue to institutions and investors than to employed clinicians [7]. To mitigate these risks, radiologists must be actively involved in AI procurement, workflow design, and implementation oversight. Governance structures should ensure transparent accountability, equitable distribution of benefits, safeguards against deskilling, and mechanisms to prevent algorithmic overreach or managerial misuse of AI-derived metrics.

## Conclusion

AI is not a remedy for burnout by default. Its effect on physician well-being depends on context, design, and institutional framing. Radiology, as the specialty with the highest AI adoption, offers an early warning system. Without deliberate efforts to align AI deployment with professional values, human factors, and systemic reform, AI may intensify rather than alleviate burnout. Further empirical research, participatory implementation models, and governance frameworks are essential to ensure AI supports rather than undermines the workforce. AI should therefore be evaluated not only for diagnostic accuracy and efficiency, but also for its structural impact on professional autonomy, accountability, and the normative conditions of clinical work.
